# Designing a patient-centered personal health record to promote preventive care

**DOI:** 10.1186/1472-6947-11-73

**Published:** 2011-11-24

**Authors:** Alex H Krist, Eric Peele, Steven H Woolf, Stephen F Rothemich, John F Loomis, Daniel R Longo, Anton J Kuzel

**Affiliations:** 1 Department of Family Medicine, Virginia Commonwealth University, Richmond, VA, USA; 2 RTI International, Research Triangle Park, NC, USA; 3 Fairfax Family Practice Centers, Fairfax, VA, USA

## Abstract

**Background:**

Evidence-based preventive services offer profound health benefits, yet Americans receive only half of indicated care. A variety of government and specialty society policy initiatives are promoting the adoption of information technologies to engage patients in their care, such as personal health records, but current systems may not utilize the technology's full potential.

**Methods:**

Using a previously described model to make information technology more patient-centered, we developed an interactive preventive health record (IPHR) designed to more deeply engage patients in preventive care and health promotion. We recruited 14 primary care practices to promote the IPHR to all adult patients and sought practice and patient input in designing the IPHR to ensure its usability, salience, and generalizability. The input involved patient usability tests, practice workflow observations, learning collaboratives, and patient feedback. Use of the IPHR was measured using practice appointment and IPHR databases.

**Results:**

The IPHR that emerged from this process generates tailored patient recommendations based on guidelines from the U.S. Preventive Services Task Force and other organizations. It extracts clinical data from the practices' electronic medical record and obtains health risk assessment information from patients. Clinical content is translated and explained in lay language. Recommendations review the benefits and uncertainties of services and possible actions for patients and clinicians. Embedded in recommendations are self management tools, risk calculators, decision aids, and community resources - selected to match patient's clinical circumstances. Within six months, practices had encouraged 14.4% of patients to use the IPHR (ranging from 1.5% to 28.3% across the 14 practices). Practices successfully incorporated the IPHR into workflow, using it to prepare patients for visits, augment health behavior counseling, explain test results, automatically issue patient reminders for overdue services, prompt clinicians about needed services, and formulate personalized prevention plans.

**Conclusions:**

The IPHR demonstrates that a patient-centered personal health record that interfaces with the electronic medical record can give patients a high level of individualized guidance and be successfully adopted by busy primary care practices. Further study and refinement are necessary to make information systems even more patient-centered and to demonstrate their impact on care.

**Trial Registration:**

Clinicaltrials.gov identifier: NCT00589173

## Background

Clinical preventive care can be highly effective at extending and improving the quality of life [1-5], but patients receive only half of indicated preventive services [6]. A host of patient, clinician, and health care system barriers exist [7-9]. Patients may lack knowledge about needed services, have limited motivation to receive services, or face logistical challenges. Clinicians may fail to address needed services due to oversight, lack of time, and competing demands. The healthcare system is fragmented and there are few tools and little infrastructure to support both clinicians and patients. To a large extent, the typical system for delivering preventive care is reactive, relying on patients to schedule wellness visits and clinicians to recognize when preventive care is due. As a result, the patients most in need of preventive care are often seen only for sick visits.

A new generation of personal health records (PHRs) has the potential to introduce a more proactive dynamic to health promotion and disease prevention. These technologies have the ability to empower patients with information, alert them when preventive services are due, and help them implement guidelines--but only if they are properly designed, adopted, and implemented. To address delivery barriers, these new PHRs will need to move beyond a record keeping functionality to collect, interpret, and translate medical information for patients. Additionally, PHRs will need to help patients and clinicians take action based on the information. Accordingly, national movements aimed at transforming healthcare delivery, including the patient-centered medical home, meaningful use, the Health Information Technology for Economic and Clinical Health (HITECH) Act, and the Affordable Care Act, advocate significant advances in the functionalities of PHRs [10-13].

### The Evolution of PHRs

Patients collecting and retaining paper copies of their immunizations and medical records can be viewed as the earliest form of a PHR. With the advent of the computer age, these paper-based systems evolved into electronic formats [14]. Websites and software products emerged that allowed patients to type in their medical information. The next advance in PHRs was to interface with electronic information to access clinical data that patients either do not know or cannot recall accurately. Some systems were "stand alone," extracting information from claims and insurer data or external registries [15,16]. Other systems were "integrated," being connected directly to the electronic medical record (EMR) of the patient's clinician and potentially allowing a bidirectional flow of information [17,18]. Linking to records allowed the PHRs to show patients lists of medications, diagnoses, laboratory results, and test dates. Now, PHRs are evolving to promote clinician patient communication through secure messaging, which is highly desired by patients [19]. It can allow patients to ask questions and clinicians to send simple responses, instructions, reports, clinical data, and reminders. Some PHRs also provide basic recommendations about when patients need preventive and chronic care based on when they last received a service; and to deliver educational materials to the patient over the web.

These PHR advances have been very beneficial to patients, but the technology allows more to be done, particularly to promote the delivery of preventive care. For example, a messaging service that allows patients to communicate with clinicians is a reactive feature, whereas current technology can be proactive in giving patients instant access to comprehensive recommendations and information about priorities for improving their health that may have escaped their attention.

Showing patients their medical information from clinical datasets and EMRs can help patients and clinicians reach a common understanding of diagnoses, medications, and results [20,21]. However, much of the information and clinical terminology needs translation into lay language. Diagnoses generated through an administrative claims process may even be phrased as arcane ICD-9 codes. Medications as well as laboratory, radiology, and procedural tests may be labeled for clinicians and not patients (e.g. fecal occult blood test rather than stool test for colon cancer screening or "2 PO BID" rather than "take 2 pills twice daily by mouth").

Displaying clinical information without interpretation or context does not inform patients about what they need to do to improve their health. Some PHRs are beginning to provide basic interpretation [22], as seen by providing laboratory reference ranges. While useful for some values, many laboratory reference ranges are incorrect for individual patients. An example is the automated labeling of low density lipoprotein cholesterol values of greater than 100 mg/dL as abnormal, which only applies to patients with coronary artery disease or an equivalent risk.

Other PHRs allow practices to define the age and frequency they wish patients to receive services; the PHR then shows patients lists of when they received a test and when it is due next based on rules defined by the practice. This can overly simplify complex national guidelines and it is inefficient for practices to individually recreate prevention rules. Many recommendations need to be personalized to each patient's complex history (e.g. family history, prior results, and abnormal values); generating recommendations merely on a patient's age and gender will make recommendations appear less relevant for patients. Additionally, there is uncertainty about some recommendations and conflicts between different organizations' recommendations. This uncertainty and conflict needs to be presented to patients and clinicians in a manner that can allow them to decide what is right for them [23].

Finally, to promote delivery of preventive services, PHRs should provide support to allow patients and clinicians to act on recommendations. The specific support needed varies depending on each patient's circumstances as well as local resources but may include educational materials, decision aids, risk calculators, logistical support, and reminders. All of these features could be programmed into PHRs.

We sought to create and test a patient-centered PHR to more deeply engage patients in preventive care and health promotion, overcoming many of the barriers described above. The PHR that we developed is an interactive preventive health record (IPHR). Ongoing studies are evaluating its impact on outcomes. This manuscript describes the design and development of the IPHR and its use by patients and practices.

## Methods

The IPHR was created over a series of three research studies - a randomized controlled trial to test whether patients mailed an invitation to use the IPHR increased delivery of preventive services (Efficacy trial, 2007-2010) and two prospective time series analyses to assess the reach, effect, adoption, implementation and maintenance of the IPHR as it was fielded to an entire primary care practice population (Adoption trial, 2010-2012) and to a diverse range of primary care practices (Dissemination trial, 2009-2011) [24,25]. The Efficacy trial was conducted in 8 practices on a subset of their patients to determine if the IPHR increased the delivery of recommended preventive care. The Adoption trial observed whether these eight practices could extend the IPHR to all adult patients utilizing existing primary care workflow and resources and whether similar the benefits seen in the Efficacy trial would be replicated. The Dissemination trial extended the IPHR to an additional 6 practices, selected to represent a range of primary care settings, to see if use of the IPHR could be generalized across settings. These latter two studies were designed to assess the generalizability and scalability of the IPHR.

To ensure that the IPHR's design was compatible with primary care practice workflow and met users' needs, extensive patient and clinician input was obtained throughout development and implementation. During early development in the Efficacy trial, usability tests were performed with 30 patients to solicit input on content, layout, and comprehension of material. Workflow was observed at the study sites. Clinicians, nurses, and staff were interviewed to determine what tools they needed to help deliver preventive care. Four patient focus groups and two practice clinician and staff focus groups provided additional insights on the IPHR's design at the end of the trial. Based on these findings, the IPHR was further revised for the Adoption and Dissemination trials. Clinicians, nurses, and staff participated in seven learning collaboratives at each site to define their prevention delivery workflow before IPHR implementation, modify their workflow to incorporate the IPHR, and share successes and challenges during the IPHR implementation phase. Patients were provided a mechanism to send feedback and content requests to the IPHR developers as they used the system. Throughout these processes the IPHR was continually improved, collectively resulting in both patients and clinicians shaping the IPHR's design.

**Figure 1:**
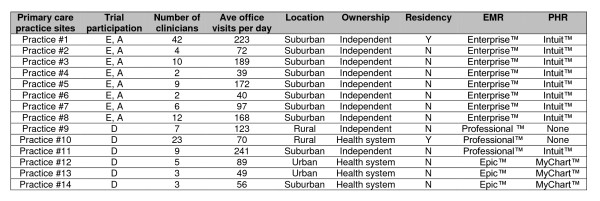
**Characteristics of the Primary Care Practices Participating in the Efficacy, Adoption, and Dissemination Trials**. E = Efficacy trial, A = Adoption Trial, D = Dissemination trial.

Fourteen primary care practices in the Virginia Ambulatory Care Outcomes Network (ACORN) with varying locations, patient populations, information systems, informatics experience, and organizational culture participated in the three trials (Figure 1) [26]. Practices were selected to represent a spectrum of typical primary care practices. The practices ranged in size from 2 to 42 clinicians (mean 9.8 clinicians) and were distributed throughout the state of Virginia (2 rural, 2 urban, and 10 suburban). Four practices belonged to larger health systems while the others were independent practices. The practices used the Enterprise™, Professional™, and Epic™ EMRs. Two practices had no PHR, 9 used Intuit™, and 3 used the MyChart™ PHR. The IPHR was integrated into MyChart™ as an application, but fielded in parallel to Intuit™ (see "Establishing an Account," below).

In addition to reporting on the IPHR design and development, this manuscript reports patient use of the system during the first six months of the Adoption and Dissemination trial (Nov 2010 - May 2011). Demographic data on all patients seen in the practices were obtained from the practices' EMR. Similar demographic data about patients who used the IPHR and the dates they used the IPHR were obtained from the IPHR database. Patient use of the IPHR was defined as the percent of patients, age 18 to 75 years, who had an office visit after the practice adopted the IPHR (denominator) and who used the IPHR to receive prevention recommendations (numerator). How patients used the IPHR (number of pages viewed and length of time viewing the IPHR) was tracked in aggregate for all patients, using Google Analytics.

## Results

### Technical Advance

The IPHR design is based on a conceptual model to make information technology more patient-centered [27]. The model specifies five necessary components which include: (1) collecting patient information, (2) integrating existing clinical data, (3) interpreting patient information, (4) providing personalized recommendations, and (5) facilitating patient and clinician action. How these components were operationalized is described in greater detail below.

**Figure 2:**
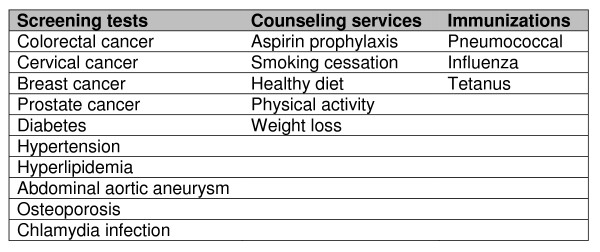
**Preventive Services Addressed by the IPHR**.

The IPHR addresses 18 clinical preventive services and their associated chronic conditions (Figure 2). Preventive services that have received an "A" or "B" recommendation from the U.S. Preventive Services Task Force (USPSTF) and that were prioritized by the National Commission on Prevention Priorities were selected for inclusion in the IPHR. The IPHR also addresses some of the chronic care recommendations associated with the USPSTF recommendations. For example, in addition to addressing screening for high cholesterol, the IPHR addresses managing high cholesterol.

### Establishing an Account

We designed the IPHR to function in a wide range of primary care practices, including practices with diverse information technology infrastructure. Accordingly, the IPHR can function as a stand-alone system dedicated to prevention (as in the study practices with no PHR or Intuit™) or integrated into a practice's existing PHR with administrative functionality and secure messaging (as in the practices using MyChart™). The stand-alone version is web-based: clinicians direct patients to http://www.MyPreventiveCare.org and provide them with an individual identification (ID) number to establish an account. The ID number is required to allow the IPHR to connect to secure clinical information residing in the EMR (see "Information Sources," *below*). In the PHR-integrated version, patients use their existing PHR and click on the IPHR link, which launches the IPHR with their ID encrypted for a seamless single sign-on experience.

### Information Sources

**Figure 3:**
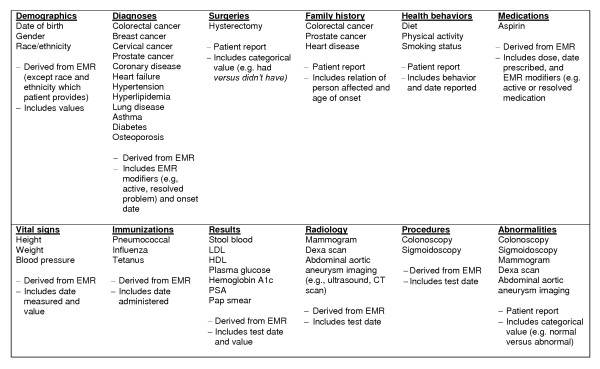
**Minimum Clinical Dataset Required by IPHR to Generate Personalized Prevention Recommendations**. The above elements are necessary to determine applicability of U.S. Preventive Services Task Force recommendations. CT = computed tomography, HDL = high-density lipoproteins, LDL = low-density lipoproteins, PSA = prostate specific antigen.

Once a patient establishes an IPHR account, the IPHR makes an open data base connection (ODBC) to the EMR of the patient's personal clinician and extracts all relevant and available clinical data (see Figure 3). These data elements represent standard clinical elements from patients' records. Accordingly, the IPHR can access this information from any electronic clinical data source. Patients are then shown their history, medications, immunizations, test dates, and results that relate to preventive care; and patients are asked to review, correct, and update their information.

Next, the IPHR administers a brief health risk assessment to ask patients about information that is not entered well electronically into EMRs or for which patients are the ultimate authority (e.g., health behaviors and psychosocial measures). Specifically, the IPHR's patient health risk assessment includes three questions about health behaviors and 9 questions about race/ethnicity, family history, and whether the patient previously had an abnormal pap smear, mammogram, or colonoscopy/sigmoidoscopy. The study sites reported not recording race/ethnicity in their EMR. The EMRs used by the study sites did not make it easy for clinicians to record family history with the necessary specificity to make screening recommendations (e.g. having a first degree relative with colorectal cancer diagnosed before the age of 60 years). While the IPHR could determine if other preventive results were normal or abnormal from the EMR data, findings from pap smears, mammograms, and biopsy reports from colonoscopies/simoidoscopies were recorded as text that the IPHR could not electronically interpret.

### Generating Personalized Recommendations

Next the IPHR makes personalized recommendations, based on nationally endorsed, evidence-based guidelines and relevant patient characteristics. The IPHR recommendations rely primarily on USPSTF guidelines but also incorporate recommendations from the Joint National Committee on Prevention, Detection, Evaluation and Treatment of High Blood Pressure (JNC VII) [28,29], the National Cholesterol Education Program (NCEP-ATP III) [30,31], the American Diabetes Association (ADA) [32], the Advisory Committee on Immunization Practices (ACIP) [33], Healthy People [34], and the Dietary Guidelines for Americans [35]. These guidelines are used because the USPSTF defers to them for guidance on immunizations (e.g. ACIP) or diagnosis and management (e.g. JNC VII, NCEP, or ADA), or because they set relevant national health behavior goals.

For some USPSTF recommendations, there is inconclusive evidence to tell patients whether they should receive a service, yet these are highly utilized services and a decision needs to be made about whether to deliver the service (e.g., whether to screen for prostate cancer or the age to start mammograms) [36]. Additionally, there are differences between some organizations' recommendations (e.g., USPSTF versus ADA's recommendation on who to screen for diabetes). The IPHR addresses these issues by identifying patients for whom there are uncertain or discrepant recommendations and promoting shared decision-making by explaining the issues, presenting how to make an individual choice based on personal risks and values, and providing decision aids and educational materials to aid in the decision-making process.

**Figure 4:**
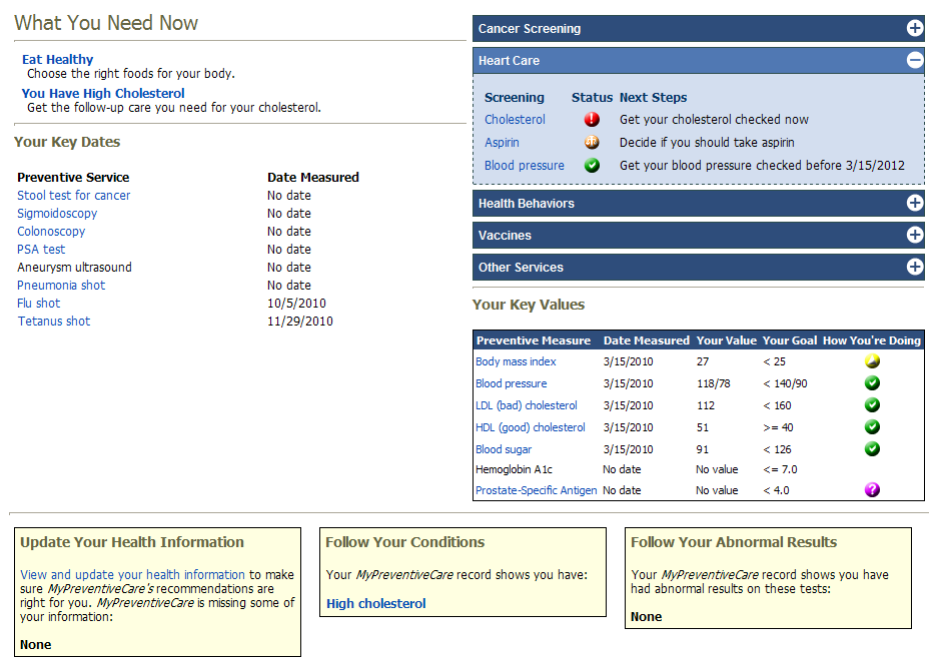
**The IPHR General Summary Page**. After completing the health risk assessment patients are directed to the IPHR general summary page. This page is intended to both provide patients an overview of how they are doing and allow them to access detailed personalized messages about any prevention top by simply clicking on the blue hyperlinked topics.

After the IPHR makes its determination of the patient's prevention status, it presents the patient an overview on a general summary page (Figure 4). This represents a snapshot of (a) what a patient needs now (i.e., clearly overdue preventive services or uncontrolled chronic conditions), (b) dates when preventive services were last received, (c) values from previous screening and monitoring tests, (d) categorical overviews of preventive care (e.g., cancer screening, heart care, health behaviors, vaccines, and other services), and (e) missing information. Recommendations are worded as simple statements and linked to visual status cues.

**Figure 5:**
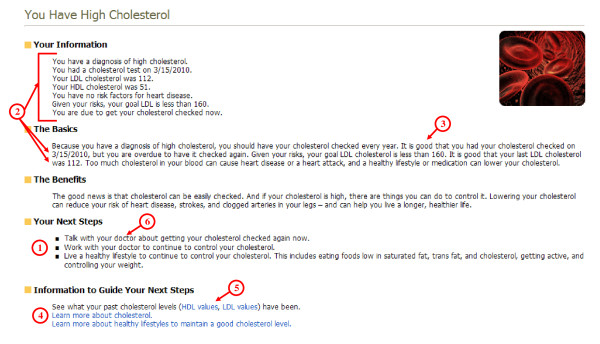
**Detailed Personal Prevention**. This is an example of a patient's detailed personal message about cholesterol. Content is modeled after HealthFinder.gov and framed to promote patient action. Specific elements include: (1) An explicit guideline-based recommendation presented in patient language; (2) Content is personalized for each patient, both summarizing the patient's individual profile and highlighting patient characteristics to make the content more relevant; (3) Positive aspects are emphasized to make the message motivational; (4) Patients are provided a personalized library of non-commercial, evidence-based, and patient-centered tools to guide their next steps and manage their preventive care; (5) Patients can view all available past values, which are graphically displayed and trended to demonstrate any changes; and (6) All content is framed to link the patient back to their personal clinician. For preventive services with a balance of risks and benefits, messages also contain (a) a description of the risks of the preventive services and (b) information about how to decide if the preventive service is appropriate for an individual.

Patients are encouraged to click on any summary page item to reach a more detailed, personalized message about the preventive service (Figure 5). The wording of the messages is modified from language developed by the U.S. Department of Health and Human Service's website, HealthFinder.gov. The messages cover five domains: a summary of the patient's information (dates, values, risks, and goals), basic information about the condition, benefits of the preventive service, next steps based on the individual patient's profile, and information to guide next steps selected based on the patient's profile.

### Making Information Actionable

As reflected in our conceptual model, a key feature of the IPHR is to help patients take action to receive preventive care. Six specific features promote and reinforce action (Figure 5). (1) Recommendations are explicit. Both action statements on the summary page and next steps on the detailed personal message provide explicit, individually tailored recommendations on what a patient should do to improve their health. (2) Content is personalized. Detailed messages are derived from each patient's individual clinical profile and personal content is embedded throughout each detailed message to add further relevance and importance to the service. (3) Content is motivational. Messages highlight positive aspects of health and concretely show the benefits of making changes. (4) Self-management tools, decision aids, links to community resources, and logistical support are provided. Each message has a personalized list of additional resources to guide the patient's next steps. These resources are selected based on the patient's anticipated needs from existing sources that are non-commercial, evidence-based, consistent with guidelines, and patient-centered. (5) Historical information is presented. Information to guide the patient's next steps includes links to available prior test results, trended and graphically displayed to highlight changes over time. (6) Care is coordinated with the patient's personal clinician. After the patient uses the IPHR, a summary is transmitted directly to the EMR of the patient's clinician, listing the patient's updates/corrections, health behaviors, and overdue preventive and chronic care. This allows the IPHR to create a shared prevention agenda for the patient and clinician.

### IPHR Use Over Time

The IPHR is intended to function as a longitudinal record and reminder system for patients and clinicians. The IPHR automatically re-queries the EMR to assess if patients are overdue for services, updates the patient's record, and generates patient email reminders and clinician EMR summaries if the patient needs a service. When patients revisit the IPHR, the general summary page reflects the most recent values and dates. Patients can continue to access past values and trends through their detailed personal prevention recommendation pages. This essentially makes the IPHR a sophisticated, longitudinal, personalized prevention plan that evolves in parallel with the patient's record [10].

### IPHR Use by Patients and Practices

**Figure 6:**
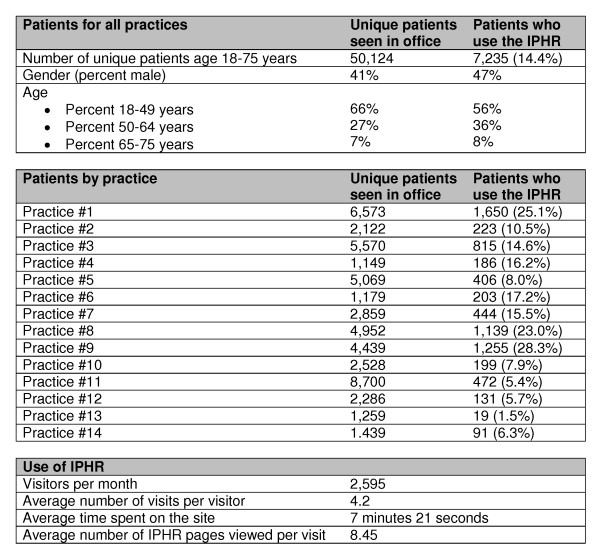
**Patient and Practice Use of the IPHR During the First Six Months of Availability**.

The IPHR was generally well accepted by primary care practices and patients. The integration of the IPHR into the practices' information systems was successful at all sites including extracting patient data from the practices' EMRs, sending summaries back into the EMRs, and integrating with the existing PHRs. Data from the 14 practices' electronic medical records indicate that 50,124 unique eligible patients had an office visit during the 6-month observation period, of whom 7,235 (14.4%) established an IPHR account and received preventive recommendations. The percentage of patients who used the IPHR ranged from 1.5% to 28.3% across the 14 practices. The patients who self selected to establish IPHR accounts were slightly older and more likely to be male then the general population of patients seen by the practices but were otherwise representative of the general primary care population (Figure 6). Of the patients who established an IPHR account, 49% and 10%, respectively, made at least one return visit to the site 0-3 months and 3-6 months after creating their IPHR account.

Data collected by web-tracking software indicate that the IPHR received 2,595 visitors per month. The average visitor spent 7 minutes 21 seconds on the site and viewed 8.45 pages on the IPHR website per visit. The majority of patient comments when using the IPHR were positive. Patients expressed that the system was easy to use ("Great program! Very user friendly, thank you."); they liked seeing their health information ("It is great to go to the site and see test results."); and they believed that the IPHR recommendations helped them to manage their preventive needs ("I see that I still have some work to do, but am pleased with the progress I have made since my last physical"). Only three patients asked to have their accounts deleted because of privacy concerns. Negative comments included a desire to see more information ("I'd like to be able to see all of my lab results") and an expression of frustration when results that were only available in hard copy were not accessible. One patient, whose labs were scanned into the system rather than transmitted electronically said, "My test results are three years out of date. I need to see my most recent information." During pre-IPHR implementation learning collaboratives, practices were concerned about the extra work required to persuade patients to use the system and to deal with incoming messages. However, after fielding the IPHR, the practices reported that patients liked the system, it was easy to explain to patients how to use the IPHR, and it helped them to deliver care. Specifically, practices reported using the IPHR to help patients prepare for visits, augment health behavior counseling discussions, better explain lab results to patients, assist with population management, remind patients when they were due for services, prompt clinicians about overdue care, and fulfill Medicare annual wellness visit requirements.

## Discussion

The technical advance of the IPHR demonstrates how to make health information technology more patient-centered [27]. By addressing patient, clinician, and system barriers, the IPHR has great potential to effectively increase the delivery of recommended preventive services. The IPHR helps patients to access, understand, and act on their clinical preventive information whenever they like. It can serve as a supplement to clinical encounters or assist in automating and personalizing population level care. Specifically, the IPHR is designed to increase patient knowledge of preventive guidelines and what national organizations specifically recommend for them; catalyze and prompt patients and clinicians to discuss needed care; prepare patients for clinical encounters and preventive service decisions; and automate the provision of educational materials, risk calculators, and decision aids with a depth of content that clinicians are unlikely to be able to provide.

Technologies that bring information to patients are increasingly recognized as important and nowhere are the opportunities greater than for prevention. Unlike questions about specific diseases that affect only subgroups of patients with the condition, the questions surrounding health promotion and disease prevention affect almost everyone, so the audience is huge [37]. Prevention has well defined guidelines that can be converted to logic within PHRs and provide patients very specific and personalized recommendations [1]. Health promotion focuses on behavior change, for which patients need substantial information and support, much of it difficult to get from clinicians. Disease prevention (screening, immunizations) requires decisions about whether to get the service, how often, and the downsides of the service. Thus, there is a great unmet need for information on these topics.

The delivery of preventive services should be a joint effort between clinicians and patients. Patient uptake is highly influenced by clinician recommendations and patients must be interested and enthusiastic about the services and changes, so both entities must participate in the delivery process [38,39]. Additionally, many preventive services have a close balance of benefits and harms, personalized recommendations based on individual risk assessments coupled with patient education is necessary [23]. PHRs can assist clinicians to more objectively and systematically provide education and prepare patients to participate in a shared decision-making process. These factors underscore the advantages of a shared tool like an integrated PHR-EMR and the inherent limitation of stand-alone PHRs or relying on solely clinical encounters.

Both the HITECH ACT and the United States' first National Prevention Strategy have laid out a road map to transform health care through the meaningful use of health information technology [40,41]. The broad strategic goals to achieve this transformation include capturing and sharing clinical data, empowering individuals to improve their health, and improving care and population health. The PHR advances advocated in this article are necessary elements of this proposed national transformation.

The needs for such PHR advances are further reinforced by the new Medicare requirements for annual wellness examinations, which require clinicians to conduct a preventive health risk assessment and provide patients a personalized 10-year prevention plan [10]. These requirements have the potential to improve health outcomes for Medicare beneficiaries, yet most clinicians cannot systematically provide patients such an assessment or plan. Tools such as the IPHR are needed to support clinicians.

The limitations of the IPHR deserve consideration. Technology cannot serve as a substitute for clinicians and the system is meant to supplement encounters and practices' population health activities. The IPHR could address more topics than the current 18 preventive and chronic care services. While recommendations are personalized on domains dictated by the guidelines, the content and presentation could be further personalized on race/ethnicity, culture, age, gender, literacy and numeracy, preferred language, socio-economic status, and geographic location. Even though some tools and resources are embedded in the detailed personal messages, the IPHR could be used to further facilitate and coordinate collaborative care between primary care clinicians, specialists, ancillary services, and community resources, making the system much more action oriented.

## Conclusions

The preliminary use of the IPHR reported in this paper offers encouragement that the IPHR and similar patient-centered information systems might be generalizable and scalable to a wide range of primary care practices. The findings derive from 14 practices in Virginia, and further research is needed to replicate these findings elsewhere. Additionally, outcomes data are needed to determine the impact of the IPHR on the delivery of care and on patient engagement in decision-making. Future manuscripts that detail the findings of our Efficacy, Adoption, and Dissemination trials will contribute to this evidence.

The ultimate goal of transforming our information systems is to improve the delivery of care and the health of patients. PHRs can play a pivotal role in helping to engage, inform, and motivate patients. While significant advances have been made in the design, adoption, and implementation of PHRs, much more is needed.

## Authors' contributions

All authors read and approved the final manuscript.

AHK - created and designed the reported technical advance, drafted and revised the submitted manuscript, and gave final approval of the published manuscript. EP - created, designed, and programmed the reported technical advance, participated in drafting and revising the submitted manuscript, and gave final approval of the published manuscript. SHW - participated in the creation and design of the reported technical advance, participated in drafting and revising the submitted manuscript, and gave final approval of the published manuscript. SFR - participated in the creation and design of the reported technical advance, participated in drafting and revising the submitted manuscript, and gave final approval of the published manuscript. JFL - created, designed, and integrated the reported technical advance into EMRs, participated in drafting and revising the submitted manuscript, and gave final approval of the published manuscript. DRL - participated in the design of the reported technical advance, participated in drafting and revising the submitted manuscript, and gave final approval of the published manuscript. AJK - participated in the design of the reported technical advance, participated in drafting and revising the submitted manuscript, and gave final approval of the published manuscript.

## Pre-publication history

The pre-publication history for this paper can be accessed here:

http://www.biomedcentral.com/1472-6947/11/73/prepub
